# An alternative method for SARS-CoV-2 detection with use modified fluorescent in situ hybridization

**DOI:** 10.1186/s13568-024-01726-z

**Published:** 2024-06-06

**Authors:** Agnieszka Sroka-Oleksiak, Agnieszka Krawczyk, Katarzyna Talaga-Ćwiertnia, Dominika Salamon, Monika Brzychczy-Włoch, Tomasz Gosiewski

**Affiliations:** 1 Department of Molecular Medical Microbiology, Chair of Microbiology, Faculty of Medicine, Jagiellonian University Medical College, 31-121 Krakow, Poland; 2 Microbiome Research Laboratory, Department of Molecular Medical Microbiology, Chair of Microbiology, Faculty of Medicine, Jagiellonian University Medical College, Czysta 18, 31-121 Krakow, Poland

**Keywords:** SARS-CoV-2, Fluorescent in situ hybridization, Viral diagnostics, Hybridization probe

## Abstract

The real-time reverse-transcriptase polymerase-chain-reaction (rRT-PCR) tests are the gold standard in detecting SARS-CoV-2 virus infection. However, despite high sensitivity and specificity, they have limitations that in some cases may result in false negative results. Therefore, it is reasonable to search for additional tools that could support microbiological diagnosis of SARS-CoV-2. The aim of the study was to develop a highly specific molecular test capable of detecting and visualizing SARS-CoV-2 infection. A universal probe and a set of 18 specific oligonucleotides with a FLAP sequence attached to them on both sides were designed to visualize SARS-CoV-2 virus infection based on the fluorescence in situ hybridization method (FISH). FISH conditions using the developed kit were standardized on the Vero CCL-81 cell line infected by SARS-CoV-2 virus. The method was tested on 290 nasopharyngeal swabs (collected in a doublet) from patients with clinical symptoms of SARS-CoV-2. Each one swab from the doublet was subjected to RNA isolation and amplification by rRT-PCR. From the second swab, a microscopic preparation was performed for FISH. The use of the rRT-PCR allowed obtaining 200 positive and 90 negative results, while our FISH method allowed for 220 positive results and 70 negative results. The differences obtained using both methods were statistically significant (*p* = 0.008). The obtained results support the use of FISH as an additional method in microbiological diagnostics of SARS-CoV-2.

## Introduction

Currently, the gold standard in the diagnosis of Severe Acute Respiratory Syndrome Coronavirus 2 (SARS-CoV-2) is molecular method like real-time reverse transcription polymerase chain reaction (rRT-PCR), based on the amplification of the viral genetic material (Corman et al. 2020). This technique is characterized by high specificity and sensitivity, as well as the ability to detect infection at an early stage. The highest sensitivity of genetic tests is observed 4–5 days after the onset of symptoms (Binny et al. 2022). Despite many advantages, rRT-PCR also has its limitations. Mistakes made during sample collection, storage or transport, as well as the appearance of new virus variants, may result in false negative results. Moreover, there is a possibility of a questionable result that requires repeating the test, which extends the diagnostic time. It is also possible for long-term persistence of positive results, despite the disappearance of clinical symptoms of COVID-19 (Rahbari et al. 2021).Other methods currently used in laboratory testing for COVID-19 include antigen and serological tests (Zheng et al. 2022). Antigen tests enable the detection of SARS-CoV-2 protein antigens. Their main advantage is the short testing time, while the disadvantages include primarily lower sensitivity in relation to genetic tests—especially in people with asymptomatic infection, which increases the risk of obtaining a negative result in a person infected with SARS-CoV-2. For this reason, each negative result obtained with an antigen test requires verification with a genetic test, especially when the patient’s clinical condition indicates SARS-CoV-2 infection. Another limitation of antigen tests is their lack of usefulness in screening tests (Di Domenico et al. 2021; Klajmon et al. 2021). In turn, serological tests involve the detection of antibodies from blood or serum samples and should be treated only as a complement to molecular methods or to assess antibody titers in recovered patients, assess response to vaccination, in epidemiological investigations, retrospective diagnostics or population studies. The use of these tests for the diagnosis of clinically active COVID-19 may be associated with obtaining false negative results (lack of seroconversion) or false positive results, caused by, among others, past infection, chronic inflammatory diseases or current infection caused by human coronaviruses (HCoVs) other than SARS-CoV-2 (e.g.HCoV-NL63, HCoV-OC43, HCoV-229E and HCoV-HKU1) or completely different viruses, as well as vaccination (Kharlamova et al. 2021; Klajmon et al. 2021). An alternative method for the diagnosis of SARS-CoV-2 may be fluorescence in situ hybridization (FISH). This molecular biology technique enables the localization, quantification, and identification of microorganisms in a biological sample. FISH directly visualizes the viral genome in infected cells (Almeida et al. 2021). This provides a more specific indication of viral presence and replication. Moreover, the high specificity of designed probes complementary to the sequence of a SARS-CoV-2 allows distinguishing it from other related viruses, which may pose a problem in antigen tests where antibodies against similar epitopes of different related viral strains may be detected. This technique can be used on a wide array of cell types provided the probe is specific to the target sequence of the genetic material. In the past, the FISH method was used in viral research, among others, in clinical trials to visualize oncolytic parvovirus after intratumoral virus administration (Kiprianova et al. 2020), or to visualization of the HIV (Vyboh et al. 2012) and EBV viruses (Lestou et al. 1996). By selecting carefully specific probes, FISH analysis provides detailed study of a specific pathogen, not only in the context of SARS-CoV-2, but also other viruses or bacteria, which may be important in the diagnosis of possible future pandemics. Therefore, it is necessary to develop new methods that could support standard diagnostics. The aim of our study was to develop the fluorescence in situ hybridization (FISH) for the direct detection of the SARS-CoV-2 in patient swabs.

## Materials and methods

### Patients

Adult patients (*n* = 290) aged 18 to 70 years with clinical symptoms of SARS-CoV-2 virus infection were recruited for the study from January to March 2021 at the University Hospital in Kraków, Poland. We included to the research all patients who presented to the hospital within a maximum of 7 days from the onset of initial symptoms of SARS-CoV-2 infection. Clinical samples were nasopharyngeal swabs. The samples were taken from patients immediately after admission to the hospital and before the initiation of any pharmacologic treatment, including antibiotic therapy. Swabs from patients were collected in duplicate (*n* = 580). One swab from the doublet was intended for viral RNA isolation and then amplification using the qPCR method. In turn, microscopic preparations were made from the second swab and the hybridization process was carried out, followed by visualization in a fluorescence microscope. Informed consent was obtained from all participants involved in the study. All experimental protocols were approved by the Jagiellonian University Ethical Committee (No. 1072.6120.333.2020 of December 7, 2020).

### Vero cell line

The positive control constituted the Vero cell line (CCL-81), which were maintained in in Dulbecco’s modified Eagle’s medium (DMEM; Thermo Fisher Scientific, Poland) supplemented with 3% heat-inactivated fetal bovine serum (FBS; Thermo Fisher Scientific, Poland) and streptomycin (100 g/ml), penicillin (100 U/ml), and ciprofloxacin (5 g/ml) and infected with the SARS-CoV-2 virus isolate 026 V-03883 [kindly granted by Christian Drosten, Charité—151 Universitätsmedizin Berlin, Germany and provided by the European Virus Archive - Global 152 (EVAg)]. The CCL-81 not infected with the SARS-CoV-2 virus was a negative control.

### Isolation of viral RNA and rRT-PCR reaction

Samples for rRT-PCR diagnostics were placed in a NUCLISWAB standard transport medium (Innovative Biotechnology Organization Ltd., Istanbul, Turkey) and delivered in deep freeze conditions to the Department of Molecular Medical Microbiology, Chair of Microbiology, Jagiellonian University Medical College. The samples were immediately frozen at − 80 °C until RNA isolation. Immediately before RNA isolation, samples were thawed and shaken vigorously in 1 ml of sterile distilled water (free of RNAse and DNAase). Next, 400 µl of the solution were used for the isolation process to detect SARS-CoV-2. RNA isolation was performed using an automated nucleic acid extraction device CroBEE (GeneProof, Brno, Czech Republic). The obtained isolates were amplified by rRT-PCR (CFX96 thermocycler, BioRad, Hercules, California, United States) using the Vitassay rRT-PCR SARS-CoV-2 kit (Vitassay Healthcare S.L.U., Huesca, Spain) according to the manufacturer’s protocol.

### Fluorescent in situ hybridization

#### Probes design

The source gene sequences of SARS-CoV-2 betacoronaviruses (shown in Table 1) were derived from The National Center for Biotechnology (NCBI): https://www.ncbi.nlm.nih.gov/gene/. Selected sequences were aligned with other coronaviruses and human genetics regions with the use of ChromasPro ver 1.7 (Technelysium Pty Ltd) software and bioinformatics tools from the NCBI website https://blast.ncbi.nlm.nih.gov/Blast.cgi?PROGRAM%20=blastn&PAGE_TYPE=BlastSearch&LINK_LOC=blasthome (last access: 24.02.2024). The designed probes.

were later tested using MultiplePrimer Analyzer (https://www.thermofisher.com/in/en/home/brands/thermo-scientific/molecular-biology/molecular-biology-learning-center/molecular-biology-resource-library/thermo-scientific-web-tools/multiple-primer-analyzer.html last access: 24.02.2024). software in order to check whether they form dimers or if they hybridize with one another. The probes set were described in Table 1.

#### Preparation of oligonucleotides and fluorescent probe for hybridization

**Fig. 1:**
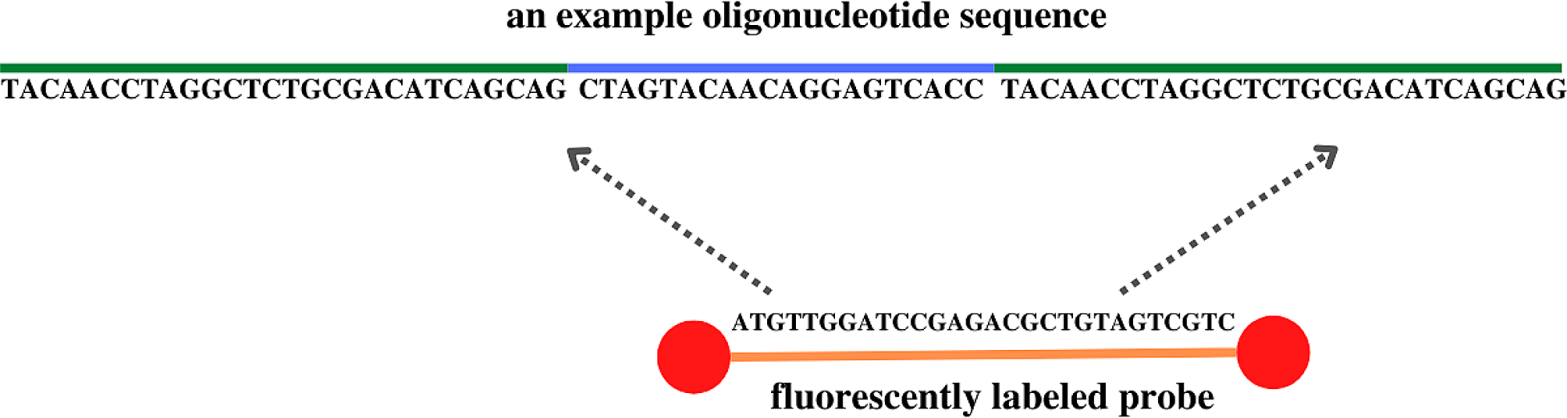
An example oligonucleotide sequence with the marked FLAP sequences and indicated probe binding site

A unique set of 18 dual-labeled oligonucleotides and fluorescent probe was designed (Table 1). FLAP sequences (green color) were attached to the oligonucleotide sequence (blue color) at both ends and they are constituted the hybridization sites for probe (orange color) attachment (Fig. 1).

Next steps were performed based on the protocol described in the publication of Rensen (Rensen et al. 2022). Tris-EDTA buffer (TE) (SigmaAldrich USA), pH = 8 was added to the synthesized oligonucleotides and the fluorescent probe (which were delivered in the form of a lyophilisate) in a volume specified for each sequence by the company (Genomed, Poznan, Poland) in order to obtain a solution with a concentration of 100µM. The fluorescent probe (100 µM) was frozen at − 20 °C until the hybridization process was carried out. Each oligonucleotide was diluted in a separate tube to a concentration of 20 µM (20 µl of oligonucleotide was mixed with 80 µl of TE). 10 µl of each oligonucleotide (20 µM) of positive polarity was added to one test tube, and 10 µl of each oligonucleotide (20 µM) of negative polarity was added to the other. As in the case of the fluorescent probe, the oligonucleotides were stored at − 20 °C until the hybridization process was carried out.

#### Preparation of buffers

*Hybridization buffer* 1 g of dextran sulfate (SigmaAldrich, USA) was mixed with 7 ml of distilled water until a homogeneous mixture was obtained. Then, 1 ml of 20 x saline-sodium citrate (SSC) solution (Ambion, USA), 1 ml of deionized formamide (Ambion, USA) were added and the volume was made up to a final volume of 10 ml with distilled water.

*Wash buffer I* 18 ml of sterile water (DNAse and RNase-free) and 2 ml of 20 x SSC were mixed in a falcon tube.

*Wash buffer II* 40 ml of sterile water (DNAse and RNase-free), 4 ml of 20 x SSC and 4 ml of formamide (Ambion, USA) were mixed in a falcon tube.

#### Preparation and preservation of microscopic specimens

150 µl of suspension containing nasopharyngeal swabs was collected and transferred to disposable cytofunnels (Thermo Scientific, UK) with microscope slides attached to them (dedicated to fluorescence in situ hybridization). The clinical material was centrifuged in a cytospin centrifuge (Thermo Shandon, cytospin 3, Thermo Scientific, UK) (200x for 4 min) and then dried at room temperature. The preparations were fixed in 4% paraformaldehyde (SigmaAldrich, USA) at 4 °C for 20–30 min. After fixation, the preparations were rinsed in a PBS solution (Ambion, USA) and then dehydrated in 70% methanol (SigmaAldrich, USA) for approximately 30 min. The slides were allowed to dry and stored in a freezer at − 20 °C until further hybridization steps were performed.

#### Hybridization

Frozen specimens were brought to room temperature and washed twice with wash buffer I at room temperature for approximately 5 min. Then, the specimens were washed once with washing buffer II for approximately 5 min. The hybridization buffer was heated to 100 °C and maintained at this temperature for 5 min to dissolve the dextran sulfate (SigmaAldrich, USA). It was then cooled to room temperature. Then, an incubation mixture was prepared to hybridize the oligonucleotides with the fluorescent probe. The reaction mixture was prepared separately for oligonucleotides of negative and positive polarity. The composition of the reaction mixture for positive polarity oligonucleotides is shown below:

- a mixture of 9 oligonucleotides with positive polarity (20 µM for each of the 9 oligonucleotides) − 2.0 µl.
- double-sided fluorescent probe (100 µM) − 1.0 µl.
- NEBuffer 3 (New England Biolabs included: 100 mM NaCl, 50 mM Tris-HCl, 10 mM MgCl2, 1 mM DTT, pH 7.9) – 1.0 µl.
- sterile water free of DNAse and RNase – 6.0 µl.

A reaction mixture was prepared analogously for oligonucleotides with negative polarity.

**Fig. 2:**
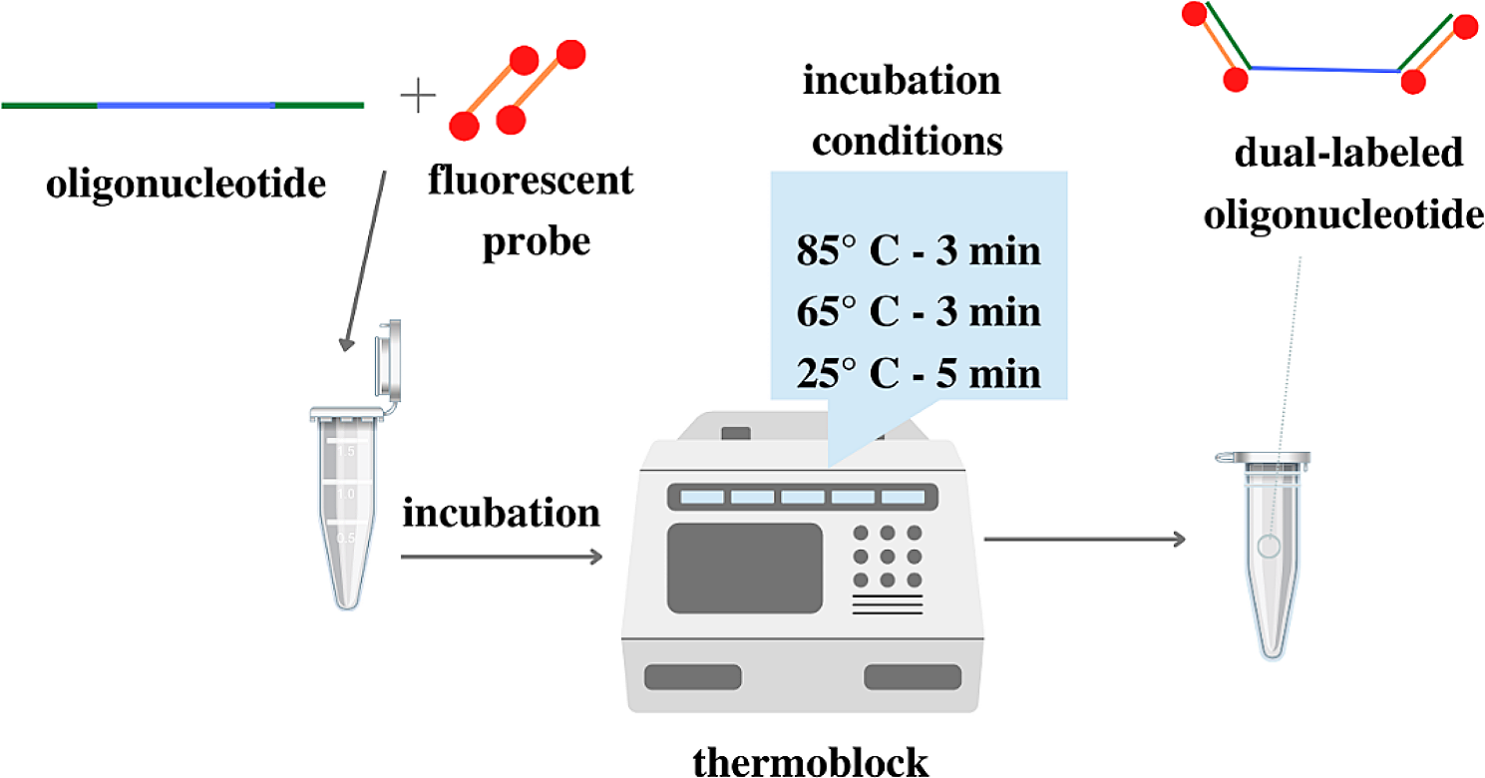
The course and preparation of the hybridization reaction

After combining the above components in test tubes, the reaction mixtures were mixed, centrifuged in a mini centrifuge (Sprout MPW-019, Adverti, Lodz, Poland) and placed in a thermoblock (Bio TDB-100, Biosan, Riga, Latvia), Fig. 2.

A mixture of probes and oligonucleotides with a total volume of 6 µl (3 µl of a mixture of positive polarity oligonucleotides and probe and 3 µl of a mixture of negative polarity oligonucleotides and probe was combined with 100 µl of hybridization buffer (volume for 1 sample) and applied to parafilm, and then covered with a microscope preparation and placed in a humid chamber. Hybridization was performed at 37 °C for 60 min without access to light.

#### Rinsing and staining

After hybridization, wash buffer II was heated in a water bath to 37 °C. Microscope slides were separated from the parafilm and incubated with wash buffer II at 37 °C for 40 min, in the dark, without mixing. Then, the microscope slides were incubated again with another portion of wash buffer II at 37 °C for 40 min in the dark, without mixing. In the next step, the microscope slides were rinsed for 5 min in a PBS solution at room temperature. A mixture of DAPI dyes (500 ng/ml, in the ratio – 4.5 µl DAPI: 45 µl of sterile water) and 0.5 µl of CellMask (dillution 1:800,000) dye for cell visualization (minimum volume for 1 slide) were applied to the slides and incubated for 5 min in the dark. The preparations were washed for 5 min in a PBS solution at room temperature. Then, the microscopic preparations were immersed in sterile water (free of DNAse and RNase), dried and stored at -20 °C until microscopic observations.

#### Imaging

Microscopic observations were performed using a BX63 Olympus microscope by performing an automatic scan of the entire preparation in 3 filters: DAPI, FITC and Texas Red (10x magnification). The images obtained from individual channels were superimposed and assessed in the context of the presence of red fluorescence in the cell cytoplasm, indicating infection with the SARS-CoV-2 virus around cell nuclei emitting blue fluorescence. Additionally, in order to control the degree and area of infection, images were assessed in the FITC channel, which allowed for the detection of cell cytoplasm stained with CellMask.

The study conducted on a group of patients was preceded by a series of experiments conducted on the CCL-81 cell infected with the SARS-CoV-2 virus (positive control) and on the Vero cell line uninfected with SARS-CoV-2 (negative control) in accordance with the methodology used to test patient samples.

#### Statistical analysis

The statistical analysis was carried out using the IBM SPSS Statistics 28. The differences between methods were analyzed using the McNemar test. The statistical significance was defined as *p* < 0.05.

## Results

### Detection of the presence of SARS-CoV-2 in Vero cells and in patients using fluorescent hybridization in situ

**Fig. 3:**
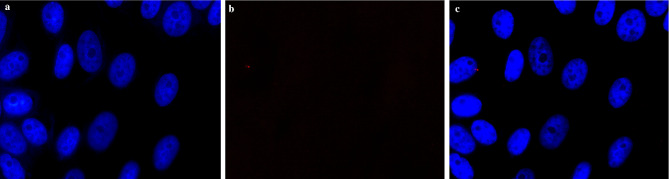
An image fragment of a uninfected cell line. Blue color (DAPI dye) visualizes cell nuclei (**a**). The absence of red color (Texas Red dye) indicates the absence of infection (**b**). The images obtained from individual channels (DAPI, FITC and Texas Red) were combined and assessed in the context of the presence of red fluorescence in the cell cytoplasm, indicating infection with the SARS-CoV-2 virus around cell nuclei emitting blue fluorescence (**c**)

**Fig. 4:**
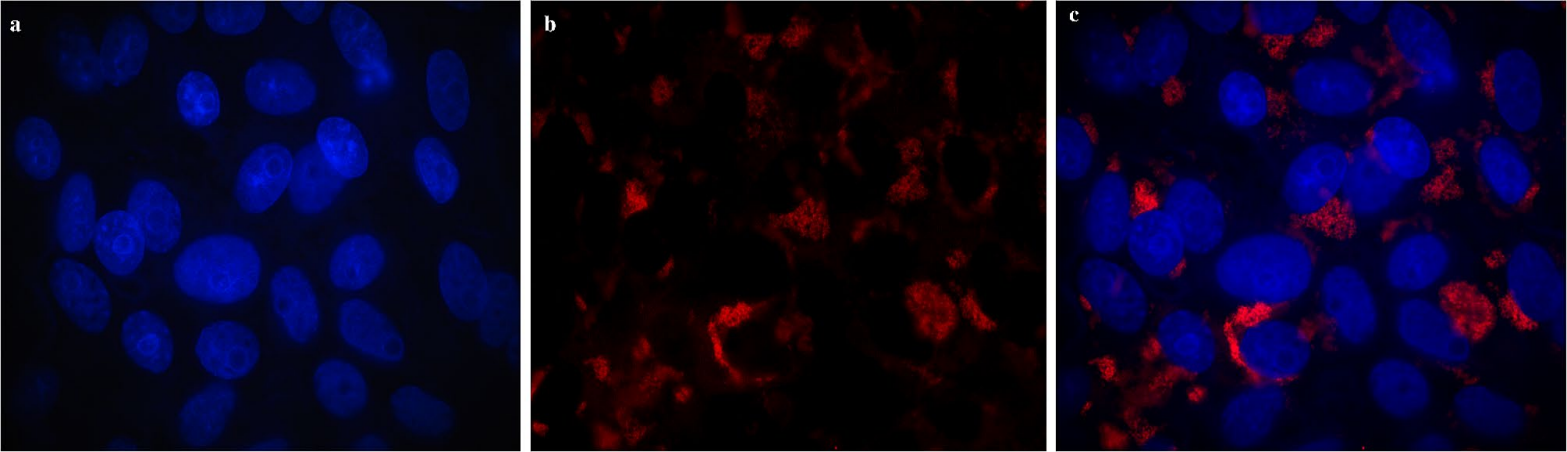
An image fragment of a cell line (Vero CCL-81) infected with SARS-CoV-2 virus. Blue color (DAPI dye) visualizes cell nuclei (**a**). The presence of red color (Texas Red dye) indicates the presence of infection (**b**). The images obtained from individual channels (DAPI, FITC and Texas Red) were combined and assessed in the context of the presence of red fluorescence in the cell cytoplasm, indicating infection with the SARS-CoV-2 virus around cell nuclei emitting blue fluorescence (**c**)

**Fig. 5:**
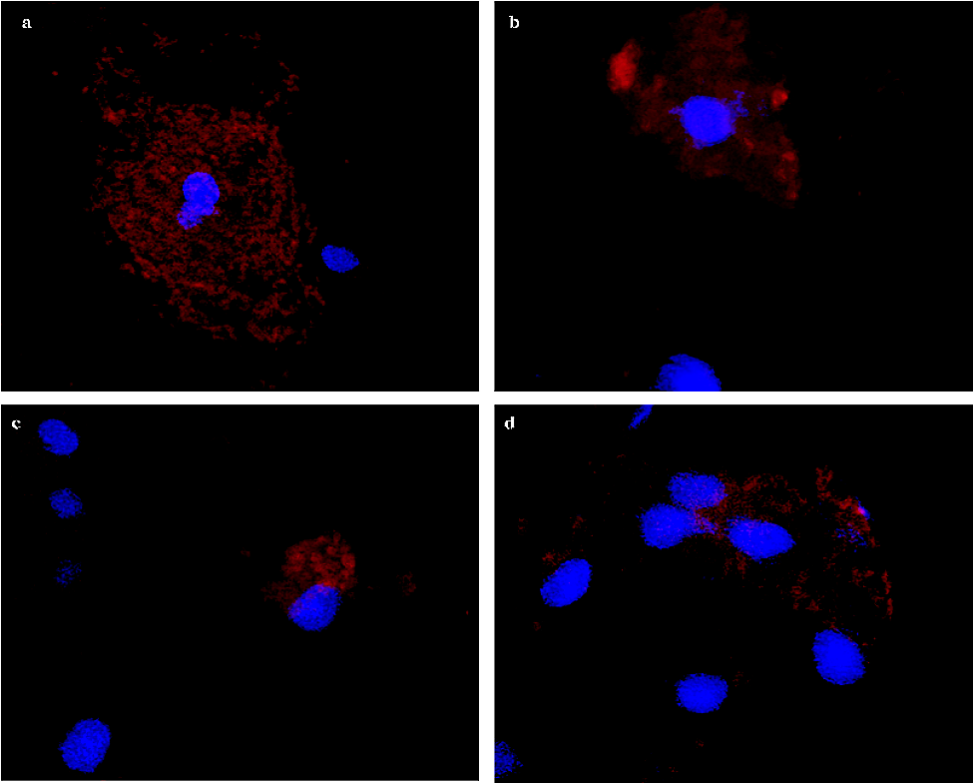
**a**–**d** Example images of clinical preparations with cells infected with the SARS-CoV-2 virus. The visible red fluorescence is derived from the replicating SARS-CoV-2 virus in the cytoplasm of cells

To test the developed probes and oligonucleotides, we first performed our methods used Vero cells. In uninfected samples, only blue fluorescence was visible (Fig. 3), indicating the cells’ nuclear DNA. In turn, in infected cells, red glowing areas were visible in the cells, indicating presence of the virus (Fig. 4). Example images of patient samples are shown in Fig. 5.

### Comparison of the FISH and rRT-PCR methods

**Fig. 6:**
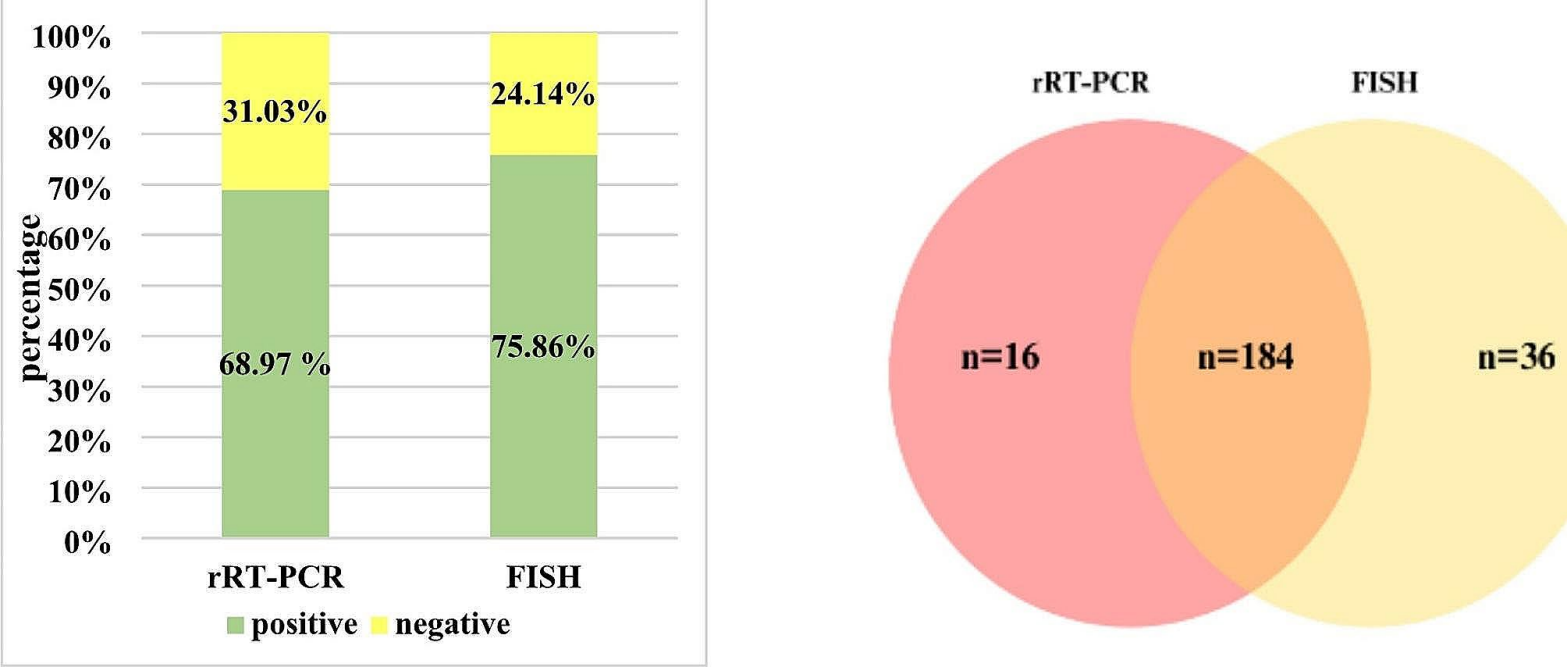
The comparison of obtained results with use of FISH and rRT-PCR methods. **a** Percentage distribution of positive and negative results. **b** Number of positive samples only in rRT-PCR (red), number of positive results both in rRT-PCR and FISH method (orange), number of positive results in FISH method only (yellow)

The use of the FISH method, allowed to obtain 220 positive (68.97%) and 70 negative results (31.03%). In comparison, the rRT-PCR method yielded 200 positive results (75.86%) and 90 negative results (24.14%) (Fig. 6a). These differences between FISH and rRT-PCR techniques were statistically significant (*p* = 0.008). Consistent results were noted for both methods in 184 positive samples and 54 negative samples. Moreover, it was observed that in 36 cases rRT-PCR was negative and FISH was positive. However, for 16 samples positive in rRT-PCR, negative FISH results were obtained, Fig. 6b.

## Discussion

In this study, we presented an alternative diagnostic method based on the detection of SARS-CoV-2 virus infection using fluorescent in situ hybridization (FISH). The essence of our method is the use of set of 18 oligonucleotide sequences with short dual FLAP sequences and hybridization probe. The oligonucleotides are selected specific fragments of the SARS-CoV-2 virus complementary to the genome of the pathogen or the transcription product. The oligonucleotides have at both ends a nucleotide FLAP sequence complementary to the labeled hybridization probe. Half of the developed oligonucleotide sequences are complementary to the sense strand and the other half to the antisense strand of the virus genome, thanks to which they can detect both the viral genomic RNA and its transcription product. This resulted in a significant improvement in detection sensitivity. We validated detection of viral RNA by fluorescence microscopy in Vero cells and then in clinical samples derived from recruited patients. The developed method allows for the detection of the genetic material of the SARS-CoV-2 virus in the tested clinical sample through in situ fluorescence visualization of virus-infected patient cells in a microscopic preparation.

Over recent years, several different FISH variants for visualizing SARS-CoV-2 RNA have been developed. Rensen et al. described smiFISH probes against the positive and negative RNA strands of SARS-CoV-2 (CoronaFISH) with highly specific viral detection in cell culture and in patient isolates (Rensen et al. 2022). Lee et al. carried out smFISH experiments with fluorescently labelled probes directed against the 30 kb viral gRNA (Lee et al. 2022). Researchers designed 48 short antisense DNA oligonucleotide probes to target the viral ORF1a and labelled with a single fluorescent dye to detect the positive sense gRNA (Lee et al. 2022). In turn, Stahl et al. established an *in-situ* hybridization technique using SARS-CoV-2 Hulu-FISH probe, MetaSystems (Stahl et al. 2021). Unlike the procedures mentioned above, our method has been modified among others, by using a set of 18 oligonucleotides designed by us along with a double-sided (complementary) fluorescent probe matched to them. Double-sided labeling of oligonucleotides with a fluorescent probe leads to a significant increase in the sensitivity of detecting infection with the SARS-CoV-2 virus in patient’s epithelial cells originating from the upper respiratory tract. Moreover, among the 18 oligonucleotides, 9 of them have a positive polarity, and the remaining 9—negative, which is important in the context of the phase of the virus replication cycle in the cytoplasm of the host cell. The genetic material of the SARS-CoV-2 virus is RNA of positive polarity, but after the virus enters the host cell, the replication stage begins and then a transcription complex is formed and, as a result, the RNA of positive polarity is transcribed into RNA of negative polarity, which will constitute a template for the replication of RNA of positive polarity (RNA (+)—the entire process takes place in the cytoplasm of cells. The inclusion of a 9 times greater number of oligonucleotides in the procedure, additionally with positive and negative polarity, makes it possible to increase the probability of SARS-CoV-2 detection regardless of the stage of the replication cycle in the host cell. Moreover, such a large number of probes complementary to different regions of the viral RNA significantly reduces the risk of failure to detect infection due to mutations in the target sequences of the SARS-CoV-2 genome—therefore, our FISH method probably will be able to detect further new variants of SARS-CoV-2.

During comparison of rRT-PCR and FISH method in our study, we observed a higher and statistically significant number of SARS-CoV-2 positive samples using FISH than in the rRT-PCR test. This is an interesting observation because in our previous studies comparing the diagnosis of bacteremia using PCR and FISH, opposite results were observed. However, it should be noted, that previous studies were based on the detection of bacteria, not viruses, and included a different procedure and hybridization probes without FLAP sequences and dual labellig. In COVID-19 diagnostics, there are also cases of false-positive results using the PCR method, but they are much rarer than false-negative (Healy et al. 2021). On the other hand, double labeling with FLAP sequences may cause the formation of non-specific bonds, which may also be the reason for the higher number of positive samples obtained by FISH compared to rRT-PCR in this study. There is a lack of publications comparing the FISH and PCR methods in microbiological diagnostics to compare our results with others, however it is quite a common topic in genetic research which show that FISH tests are more accurate than PCR assays (Belaud-Rotureau et al. 2002; Sato et al. 2003; Cox et al. 1998).

The PCR method, despite moderate sensitivity and high specificity and being recognized by the CDC and WHO as the gold standard in COVID-19 diagnosis, show a huge number of false-negative results (Rahbari et al. 2021; Lippi et al. 2020). This is due to, among others, by laboratory errors associated with sampling time, sample size, sample transfer and storage or factors during the sample preparation such as nucleic acid isolation, cDNA synthesis, and PCR amplification and with post-analytical mistakes such as interpretation and analysis of results and test report (Lippi et al. 2017, 2020; Tang et al. 2020; Espy et al. 2006). The PCR requires maintaining the continuity of the DNA/RNA sequence between both primers, which translates into the need to obtain high-quality genetic material after isolation. In the case of our FISH method, only the presence of binding sites for 18 oligonucleotides in the entire genetic material of the virus is sufficient. For this reason, the developed method is less sensitive to pre-laboratory errors and mistakes during prolonged transport of samples to the laboratory. Moreover, this method enables the visualization of cell infection with the SARS-CoV-2 virus, which translates into a reduction in the occurrence of false-positive results caused by image artifacts (visual inspection by the diagnostician). The use of one universal probe to label various oligonucleotide sequences reduces costs 18 times because there is no need to synthesize each probe separately. This method can also be applied to other high-sensitivity FISH applications. Moreover, such a large number of probes complementary to different regions of the viral RNA significantly reduces the risk of failure to detect infection due to mutations in the target sequences of the SARS-CoV-2 genome - therefore, the FISH method will be able to detect further new variants of SARS-CoV-2.

The limitation of our study is lack of calculations regarding the size of the study group. We did not determine the virus detection limit using the Vero cell line or the TCID50 or pfu virus dose. Moreover, we did not mark the degree of sequences conservatism among different viral variants and did not determine the virus detection limit. On the other hand, our goal was to increase the probability of virus detection by using 6 different oligonucleotides that attach to different places for each of the three regions. Additionally, we included polarity (positive and negative) among these oligonucleotides to be able to detect the virus both after entering the host cell (positive polarity) and after replication and formation of a transcription complex, resulting in the formation of RNA with negative polarity as previously mentioned. Therefore, the number of oligonucleotides for a given region makes it possible to increase the probability of detecting the virus despite possible mutations in a given region(s). Moreover, thanks to the 6x approach described above, we increase the probability of virus detection compared to the RT-PCR method, which uses only 1 pair of primers for the same region.

It is important to improve diagnostic methods to increase the sensitivity and specificity of detection of viral infection. The developed technique is characterized by higher sensitivity and is insensitive to pre-laboratory mistakes. Moreover, our method reduces the risk of failure to detect infection due to mutations in target sequences, so it will be able to detect new variants of SARS-CoV-2. Thanks to the appropriate design of probe sequences, FISH can also be implemented for the diagnosis of other diseases and for the detection of other pathogens, which is particularly important in the context of future pandemics.

**Table 1:** Developed oligonucleotide and probe sequences

Sequence no.	The name of the target sequence for the SARS-CoV-2 virus genome	Sequence	Polarity	GenBank Accession numbers
1	SARSCoV2_E1	TACAACCTAGGCTCTGCGACATCAGCAGCACGAGAGTAAACGTAAAAAGAAGGTACAACCTAGGCTCTGCGACATCAGCAG	Negative	*E_protein* *NC_045512.2*
2	SARSCoV2_E2	TACAACCTAGGCTCTGCGACATCAGCAGGCAAGAATACCACGAAAGCAAGTACAACCTAGGCTCTGCGACATCAGCAG	Negative	
3	SARSCoV2_E3	TACAACCTAGGCTCTGCGACATCAGCAGCTCTTCCGAAACGAATGAGTACATTACAACCTAGGCTCTGCGACATCAGCAG	Negative	
4	SARSCoV2_N1	TACAACCTAGGCTCTGCGACATCAGCAGTGCCATGTTGAGTGAGAGCGTACAACCTAGGCTCTGCGACATCAGCAG	Negative	*N_protein_* *NC_045512.2*
5	SARSCoV2_N2	TACAACCTAGGCTCTGCGACATCAGCAGGATTGCGGGTGCCAATGTGATACAACCTAGGCTCTGCGACATCAGCAG	Negative	
6	SARSCoV2_N3	TACAACCTAGGCTCTGCGACATCAGCAGAGCCATTCTAGCAGGAGAAGTTCCTACAACCTAGGCTCTGCGACATCAGCAG	Negative	
7	SARSCoV2_ORF1ab1	TACAACCTAGGCTCTGCGACATCAGCAGTTAGCCCAAAGCTCAAATGCTACTACAACCTAGGCTCTGCGACATCAGCAG	Negative	*ORF1ab_protein_NC_045512.2*
8	SARSCoV2_ORF1ab2	TACAACCTAGGCTCTGCGACATCAGCAGGTTCGAAGGCATAGCCTTCTAATACAACCTAGGCTCTGCGACATCAGCAG	Negative	
9	SARSCoV2_ORF1ab3	TACAACCTAGGCTCTGCGACATCAGCAGCTTCTCTAGTAGCATGACACCTACAACCTAGGCTCTGCGACATCAGCAG	Negative	
10	SARSCoV2_E1	TACAACCTAGGCTCTGCGACATCAGCAGACACTAGCCATCCTTACTGCGCTTTACAACCTAGGCTCTGCGACATCAGCAG	Positive	*E_protein* *NC_045512.2*
11	SARSCoV2_E2	TACAACCTAGGCTCTGCGACATCAGCAGGACAGGTACGTTAATAGTTAATAGCGTACAACCTAGGCTCTGCGACATCAGCAG	Positive	
12	SARSCoV2_E3	TACAACCTAGGCTCTGCGACATCAGCAGCGTGTTAAAAATCTGAATTCTTCTAGTACAACCTAGGCTCTGCGACATCAGCAG	Positive	
13	SARSCoV2_N1	TACAACCTAGGCTCTGCGACATCAGCAGGTTCTAAATCACCCATTCAGTACTACAACCTAGGCTCTGCGACATCAGCAG	Positive	*N_protein_* *NC_045512.2*
14	SARSCoV2_N2	TACAACCTAGGCTCTGCGACATCAGCAGGTCTGATAATGGACCCCAAAATCATACAACCTAGGCTCTGCGACATCAGCAG	Positive	
15	SARSCoV2_N3	TACAACCTAGGCTCTGCGACATCAGCAGGAAGAAGGCTGATGAAACTCATACAACCTAGGCTCTGCGACATCAGCAG	Positive	
16	SARSCoV2_ORF1ab1	TACAACCTAGGCTCTGCGACATCAGCAGCTAGTACAACAGGAGTCACC TACAACCTAGGCTCTGCGACATCAGCAG	Positive	*ORF1ab_protein_NC_045512.2*
17	SARSCoV2_ORF1ab2	TACAACCTAGGCTCTGCGACATCAGCAGGTTACATTTTTCCCTGACTTAAATACAACCTAGGCTCTGCGACATCAGCAG	Positive	
18	SARSCoV2_ORF1ab3	TACAACCTAGGCTCTGCGACATCAGCAGGTAGACGGTTGTAATTCATCAACTACAACCTAGGCTCTGCGACATCAGCAG	Positive	
19	Fluorescent probe	ROX-CTGCTGATGTCGCAGAGCCTAGGTTGTA-ROX	N/A	N/A

## Data Availability

The datasets generated during and/or analyzed during the current study are available from the corresponding author on reasonable request.

## Abbreviations

EBV: Epstein Barr virus
FISH: Fluorescence in situ hybridization
HCoVs: Human coronaviruses
HIV: Human immunodeficiency virus
rRT: PCR-real-time reverse-transcriptase polymerase-chain-reaction
SARS: CoV-2-Severe acute respiratory syndrome coronavirus 2
SSC: Saline-sodium citrate solution
